# Managing menstruation in the workplace: an overlooked issue in low- and middle-income countries

**DOI:** 10.1186/s12939-016-0379-8

**Published:** 2016-06-06

**Authors:** Marni Sommer, Sahani Chandraratna, Sue Cavill, Therese Mahon, Penelope Phillips-Howard

**Affiliations:** Mailman School of Public Health, Columbia University, 722 W. 168th Street, New York, NY 10032 USA; UNICEF Consultant, 27 Cranbrook Drive, Maidenhead, Berks UK; WaterAid, 47-49 Durham Street, London, SE11 5JD UK; Liverpool School of Tropical Medicine, Pembroke Place, L3 5QA Liverpool, UK

**Keywords:** Menstruation, Women’s health, Workplace, Low- and middle-income countries, Gender

## Abstract

The potential menstrual hygiene management barriers faced by adolescent girls and women in workplace environments in low- and middle-income countries has been under addressed in research, programming and policy. Despite global efforts to reduce poverty among women in such contexts, there has been insufficient attention to the water and sanitation related barriers, specifically in relation to managing monthly menstruation, that may hinder girls’ and women’s contributions to the workplace, and their health and wellbeing. There is an urgent need to document the specific social and environmental barriers they may be facing in relation to menstrual management, to conduct a costing of the implications of inadequate supportive workplace environments for menstrual hygiene management, and to understand the implications for girls’ and women’s health and wellbeing. This will provide essential evidence for guiding national policy makers, the private sector, donors and activists focused on advancing girls’ and women’s rights.

## Background

Despite a growing body of literature on the water and sanitation related challenges facing menstruating girls and female teachers in schools in low- and middle-income countries (LMIC) [1], evidence about adolescent girls’ and women’s menstrual hygiene management (MHM) in the workplace remains limited. For the purposes of this paper, workplace refers to any formal or informal context in which girls and women are engaged in income-generating activities, and MHM refers to the agreed upon definition (in 2012) by the Joint Monitoring Programme of WHO/UNICEF (See Table 1) [2].

**Table 1:** Definition of adequate MHM

Women and adolescent girls are using a clean menstrual management material to absorb or collect menstrual blood, that can be changed in privacy as often as necessary for the duration of a menstrual period, using soap and water for washing the body as required, and having access to facilities to dispose of used menstrual management materials. They understand the basic facts linked to the menstrual cycle and how to manage it with dignity and without discomfort or fear.	

To effectively manage their menstruation, adolescent girls and women require access to water, sanitation and hygiene (WASH) facilities, affordable and appropriate menstrual hygiene materials and services for their disposal, information on good practices, and a supportive environment where they can manage menstruation without embarrassment or stigma.

Donors and development agencies are increasingly focusing on girls and women in their efforts to eradicate poverty. Supporting women to earn a living is central to this. However, the provision of adequate, private, clean, and safe toilets, sources of water, and disposal systems, ensuring sufficient toilet breaks and defining how existing WASH inadequacies impact girls’ and women’s health and productivity in workplace environments have been neglected. We expect that many LMIC do not meet reasonable standards for WASH that are supportive of MHM in their workplaces or associated accommodations (e.g. dormitories provided for garment workers). Lack of facilities for girls and women is a rights, equity, wellbeing, and health concern, and this commentary examines the unmet needs and policy implications as a contribution to this journal’s anniversary issue.

### MHM in the workplace – status of the problem

#### A problem of size

There currently exists minimal data on how supportive of MHM workplace WASH environments are (or are not), and about the impact of workplace adequacy (or inadequacy) on adolescent girls and women. This prevents a robust understanding and evaluation of women and adolescent girls’ needs in relation to MHM in the workplace. However, data that are available indicate the size of the population facing potential workplace health inequities in relation to menstruation (and other gendered WASH-related needs) is significant. Over 800 million adolescent girls and women worldwide are menstruating on any given day [3]. Menstrual aged girls and women (~12 to 49 years) represent a significant and growing portion of the 1.2 billion women employed globally [4], with women representing nearly half of the global labor market [5, 6].

#### A problem of setting

The MHM challenges faced by working adolescent girls and women in LMIC are compounded by the nature of their work. Apart from formal workplace environments, which often may not meet adequate WASH standards that are supportive of MHM, a large number of women are employed in the informal sector. In India, 86 % of people work in the informal economy, 91 % of whom are women [7]. Lack of enforcement of occupational safety regulations and standards, if existing, add to the vulnerabilities of employees in the informal sector. For example, in some places WASH standards may exist that incorporate requirements relevant for effective MHM (such as safe, clean, private toilets with water available for washing). However employers in the informal sector may have no legal obligation to provide women with a workplace environment that is suitable for their sanitation-related needs. Compounding this challenge, girls and women in such contexts may lack the legal, social, or political power to influence inadequate enforcement of WASH standards in workplace environments. In addition, even where adequate WASH facilities that are supportive of MHM exist, women and girls may be prevented from freely using them.

In rural contexts in LMIC, women contribute extensively to the agricultural sector. Of all women working in sub-Saharan Africa and South Asia, 60 % work in the agricultural sector [4]. When these women menstruate, their workplaces pose particular challenges, such as a lack of sanitation facilities, being remotely located, or having very long workdays in the fields. These in turn preclude girls and women from having the time or privacy to attend to their MHM needs. For women working in the informal sector, including for example vendors or construction workers, their MHM sanitation-related needs may not be perceived as a priority for employers. If girls and women are self-employed workers (e.g. food vendors in a marketplace), there may be no available public WASH facilities available to them for use during the workday period.

In urban contexts, working adolescent girls and women are often forced to live, work, and travel in over-crowded spaces, affording them limited privacy and inadequate hygienic spaces for MHM. They are heavily represented in the manufacturing industry, in the service sector, and as domestic workers. One study noted women in textile factories used discarded factory cloths as MHM materials, with such rags often doused in chemicals, possibly causing irritation [3]. A study in Cambodia found that women garment workers in factories faced numerous challenges due to the limited privacy available for managing WASH needs in their associated living environments [8].

The home is also a workplace for many women and girls: it is estimated that 663 million people do not have access to improved drinking water globally and 2.4 billion people globally have no access to improved sanitation facilities. Of them, 946 million defecate in the open [9].

### Factors limiting MHM standards in the workplace

#### A problem of social norms and unvoiced needs

Women in many settings are unable to voice their right to water and sanitation, and hence to supportive environments for (menstrual) hygiene. This is grounded in women’s status in society, compounded by a lack of skills to become advocates, their fear of losing their employment, as well as physical and financial restrictions that prevent individuals from taking independent action. A key challenge to addressing the gap in attention to MHM in the workplace are existing taboos around discussing MHM at local, national and global levels [1, 10]. As the strong body of evidence on MHM in schools has demonstrated, both girls and female teachers are hesitant to mention menstruation or their MHM needs in the school environment, feeling shame, embarrassment and fear of ridicule [10].

#### A problem of advocacy

United Nations’ agencies, bilateral donors, governments, non-governmental organizations and Ministries of Education have begun advocating to diminish taboos around MHM in schools in LMIC. Such change however requires political commitment. For example, the Uganda Ministry of Education included specific attention to addressing schoolgirls’ and female teacher’s MHM needs in the National Education Policy with its own budget line item. Similarly, as the actual provision of WASH facilities is beyond the responsibility of national governments, there is a need for commitment from private sector companies, factory owners, small-scale businesses, and the agricultural sector to address this topic, despite existing taboos.

#### A problem of policy

In most LMIC, WASH guidelines and standards that explicitly support MHM (including in national occupational health or health and safety regulations) are limited or do not exist. If developed, promoted and enforced, such guidelines and standards would serve to hold businesses and governments responsible for the provision of basic private, safe, clean WASH facilities and disposal systems in workplace environments. Augmenting the barriers facing women, transportation hubs in many LMIC, such as bus stops, that are essential for commuting to work, infrequently provide safe, hygienic WASH facilities. This may hinder women’s abilities to participate in daily economic activities and leave menstruating women vulnerable to discomfort, embarrassment and potentially missed work.

### The social, financial and health consequences of not supporting MHM in the workplace

There exist significant social and financial costs for adolescent girls and women by not addressing MHM in the workplace. For some girls and women, not having a safe private location for changing used menstrual materials may lead to anxiety and stress, and in turn reduce concentration and productivity. Other girls and women may choose to miss hours or entire days of work rather than attempt to manage their menstruation in difficult environments, resulting in lower productivity. This in turn impacts their own income and that of their employers. Analyses of access to water and sanitation in the household have indicated that there exist economic benefits of WASH access from less time being sick, less money spent on medications, and less time missing school or work. As a 2012 cost-benefit analysis of the Millennium Development Goals indicated, for every one dollar spent on water and sanitation, there is a $4.3 return on investment [11]. A similar global costing focusing on the implication of unsupportive environments for MHM in the workplace has never been conducted.

However the substantive financial costs of inadequate workplace environments for MHM were highlighted in a four-country study analyzing the economic impacts of sanitation in Southeast Asia [12]. Around a quarter of all workplaces did not have toilets in Cambodia, and around 14 % of workplaces had inadequate toilets in the Philippines. In Vietnam, around three percent of health stations and 74 % of market places had no toilets and 11 and 13 % respectively had inadequate toilets [12]. Assuming women employees were absent for one day a month due to a lack of WASH facilities during their menstrual period, the study estimated 13.8 and 1.5 million workday absences in the Philippines and Vietnam respectively, with an economic loss of USD 13 and 1.28 million per year [12]. Further studies are needed on the health, economic and dignity related impacts of inadequate MHM in workplace environments.

#### The health consequences

Although only minimally focused on MHM to date, there are also documented health-related consequences of not providing adequate WASH facilities for girls and women. Whereas women and girls may withhold urination and defecation when faced with inadequate toilets, it is not possible to stop menstrual flow. When forced to change in open spaces under cover of darkness, they may be at increased risk of sexual assault [13]. There is a small literature suggesting that poor MHM affects women’s risk of reproductive tract infections and urinary tract infections [14]. Many contexts additionally lack adequate disposal facilities. Although some municipalities have rubbish waste collection systems for burning, or burial, these are mainly located in urban areas. Without such systems, disposal of absorbents may contribute to health hazards, polluting the land and rivers if freely discarded in either, and blocking toilets if available.

### Identifying opportunities and solutions

A key challenge to addressing the potential WASH and MHM-related inequities facing adolescent girls and women is determining who should take responsibility for ensuring adequate and MHM-supportive facilities in workplaces. For MHM in the school environment, the Ministry of Education is a clear lead institution. In contrast, the range and types of “workplaces” in LMIC are numerous, with businesses being diverse in size, location and scope, and many girls and women working in both the informal and formal sectors. Identifying one institutional body that will have ultimate responsibility is thus complex and potentially not an appropriate way forward. Ministries of Health and trade related ministries certainly have a role to play, particularly in establishing regulations and enforcement related to occupational health and staff welfare.

Similarly, private companies and employers also have a responsibility to prioritize this issue and take action. There has been some nascent action from the private sector already, with the CEO Water Mandate highlighting the role of the private sector in providing water and sanitation in the workplace [15]. In addition, the World Business Council on Sustainable Development is promoting a WASH at the workplace pledge to implement access to safe WASH at the workplace “at an appropriate level of standard for all employees in all premises under their control within three years after signature” [16]. In 2007, the Business for Social Responsibility through the HER project explored the MHM needs of women working in factories in Africa and Asia, aiming to improve women’s health while also improving economic productivity [17]. Subsequently, local NGOs were connected with international companies and their factories to implement programmes to increase women’s health awareness in the workplace [17].

Although national and local level solutions are needed, the International Labour Organization (ILO) offers an opportunity for spearheading effective guidance at the global level that governments can subsequently adapt (see Table 2).

**Table 2:** Relevant ILO convention and recommendations for MHM [20]

ILO Convention No 161 of 1985 on Occupational Health Services states that employers have responsibility for the health and safety of its employees including occupational functions and factors ‘*which may affect workers’ health including sanitary installations’*.	
The ILO Recommendation No. 115 of 1961 on Workers’ Housing also highlights the need for housing standards that include the supply of safe water, sewage and garbage systems, drainage and sanitary conveniences.	

In pursuing the current recommendations set forth by the ILO, those beyond national governments and the private sector have a role to play. For example, trade associations and trade unions have the potential to encourage good practice and support workers rights in this area. In LMIC, there have been small efforts to mobilize action on MHM from within trade associations; the Zimbabwe Congress of Trade Unions and Action for Southern Africa initiated a campaign in 2005 called ‘Dignity! Period’; unions in the Philippines have argued for menstrual leave for women; and in Indonesia, menstrual leave is a legal entitlement. Importantly, even in some high-income countries, unions serve an important role in pushing for such standards to be met. For example, although the United Kingdom Trades Union Congress has provided guidance to its members on ensuring better welfare facilities, including toilets, British workers have reported insufficient access to WASH facilities, employers who do not give adequate permission to use toilets, inadequate supplies of soap, toilet paper and locks on doors, and workers’ pay being reduced due to toilet breaks [18].

The notion that a basic WASH standard should be in place – so that women can urinate, defecate and manage menstrual blood flow with privacy, safety, and comfort while participating in informal or formal work in a given context – has not been specifically included in the 2030 Agenda for Sustainable Development. However opportunities exist in the Sustainable Development Goals (SDGs, See Table 3) [19].

**Table 3:** Relevant targets and goals in the SDGs for workplace MHM

SDG6 on clean water and sanitation, target 6 · 2 *’…to achieve access to adequate and equitable sanitation and hygiene for all… paying special attention to the needs of women and girls’;*	
SDG 8 on economic growth, employment and work, target 8 · 8 to *‘protect labor rights and promote safe and secure working environments for all workers, including migrant workers, in particular woman migrants and those in precarious employment.’*	

## Conclusions

Improving WASH standards that are supportive of MHM for adolescent girls and women in the workplace is beneficial to population health and economic development. It also contributes to human dignity and the attainment of human rights. Improving workplace standards so that they are supportive of MHM (and other WASH-related needs) will require the integration of guidance for MHM into existing (or new) country-level WASH standards and regulations, including into systems for monitoring occupational health and safety in the workplace. Specific recommendations include: One, the conduct of a robust costing of the potential economic losses faced by countries providing inadequate WASH for women in the workplace. This in turn might spur governments into making policies that will regulate (and enforce) the provision of gender appropriate WASH facilities in work environments, transportation hubs and other public spaces. Two, increased country-level documentation of the WASH environment for women in formal and informal work environments. Such research would provide important evidence for action at policy and programme levels. Lastly, there is a need for enhanced global advocacy about the essential importance of providing improved WASH workplace standards to support adolescent girls and women with all their sanitation needs, including MHM in LMIC. Such advocacy can serve to break taboos over discussing how to address MHM and WASH-related barriers in workplace environments, and put pressure on the private sector, national governments and international bodies to address this critical neglected issue of health inequity for adolescent girls and women.

## Abbreviations

ILO, International Labour Organization; LMIC, low- and middle-income countries; MHM, Menstrual hygiene management; SDGs, sustainable development goals; UNICEF, United Nations Children’s Fund; USD, United States Dollars; WASH, water, sanitation and hygiene; WHO, World Health Organization
